# Efficacy and tolerability of anti-programmed death-ligand 1 (PD-L1) antibody (Avelumab) treatment in advanced thymoma

**DOI:** 10.1186/s40425-019-0723-9

**Published:** 2019-10-21

**Authors:** Arun Rajan, Christopher R. Heery, Anish Thomas, Andrew L. Mammen, Susan Perry, Geraldine O’Sullivan Coyne, Udayan Guha, Arlene Berman, Eva Szabo, Ravi A. Madan, Leomar Y. Ballester, Stefania Pittaluga, Renee N. Donahue, Yo-Ting Tsai, Lauren M. Lepone, Kevin Chin, Fiona Ginty, Anup Sood, Stephen M. Hewitt, Jeffrey Schlom, Raffit Hassan, James L. Gulley

**Affiliations:** 1 Thoracic and Gastrointestinal Malignancies Branch, Center for Cancer Research, National Cancer Institute, National Institutes of Health, 10-CRC, Room 4-5330, 10 Center Drive, Bethesda, MD 20892 USA; 2 Laboratory of Tumor Immunology and Biology, Center for Cancer Research, National Cancer Institute, National Institutes of Health, Bethesda, MD USA; 3 Laboratory of Muscle Stem Cells and Gene Regulation, National Institute of Arthritis and Musculoskeletal and Skin Diseases, National Institutes of Health, Bethesda, MD USA; 4 Genitourinary Malignancies Branch, Center for Cancer Research, National Cancer Institute, National Institutes of Health, 10 Center Dr., 13N240, Bethesda, MD 20892 USA; 5 Lung and Upper Aerodigestive Cancer Research Group, Division of Cancer Prevention, National Cancer Institute, National Institutes of Health, Bethesda, MD USA; 6 Laboratory of Pathology, Center for Cancer Research, National Cancer Institute, National Institutes of Health, Bethesda, MD USA; 7 EMD Serono, Billerica, MA USA; 8 GE Global Research Center, Niskayuna, NY USA

**Keywords:** Thymoma, Immunotherapy, Avelumab, Immune-related adverse events, Immunosuppressive therapy, Anti-PD-L1

## Abstract

**Background:**

Thymic epithelial tumors are PD-L1–expressing tumors of thymic epithelial origin characterized by varying degrees of lymphocytic infiltration and a predisposition towards development of paraneoplastic autoimmunity. PD-1–targeting antibodies have been evaluated, largely in patients with thymic carcinoma. We sought to evaluate the efficacy and safety of the anti-PD-L1 antibody, avelumab (MSB0010718C), in patients with relapsed, advanced thymic epithelial tumors and conduct correlative immunological studies.

**Methods:**

Seven patients with thymoma and one patient with thymic carcinoma were enrolled in a phase I, dose-escalation trial of avelumab (MSB0010718C), and treated with avelumab at doses of 10 mg/kg to 20 mg/kg every 2 weeks until disease progression or development of intolerable side effects. Tissue and blood immunological analyses were conducted.

**Results:**

Two of seven (29%) patients with thymoma had a confirmed Response Evaluation Criteria in Solid Tumors–defined partial response, two (29%) had an unconfirmed partial response and three patients (two thymoma; one thymic carcinoma) had stable disease (43%). Three of four responses were observed after a single dose of avelumab. All responders developed immune-related adverse events that resolved with immunosuppressive therapy. Only one of four patients without a clinical response developed immune-related adverse events. Responders had a higher absolute lymphocyte count, lower frequencies of B cells, regulatory T cells, conventional dendritic cells, and natural killer cells prior to therapy.

**Conclusion:**

These results demonstrate anti-tumor activity of PD-L1 inhibition in patients with relapsed thymoma accompanied by a high frequency of immune-related adverse events. Pre-treatment immune cell subset populations differ between responders and non-responders.

**Trial registration:**

ClinicalTrials.gov - NCT01772004. Date of registration – January 21, 2013.

**Electronic supplementary material:**

The online version of this article (10.1186/s40425-019-0723-9) contains supplementary material, which is available to authorized users.

## Background

Thymic epithelial tumors (TETs, consisting of thymomas and thymic carcinomas) arise from epithelial cells of the thymus and have varying degrees of non-neoplastic immature lymphocytic infiltrations [1]. Patients with metastatic TETs have limited treatment options [2]. Thymomas are often associated with autoimmune diseases due to alterations in self-tolerance and expression of novel antigens [3].

Programmed death-1 (PD-1) is a receptor expressed on activated T cells, which, upon binding to its ligands PD-L1 or PD-L2, causes T-cell inhibition [4]. PD-1–induced T-cell anergy is important in preventing autoimmunity but can abrogate anti-tumor immune response [5]. Antibodies targeting PD-1 and PD-L1 are active against various cancers. Among these is avelumab, a fully human anti-PD-L1 IgG1 monoclonal antibody (MAb) that is approved for treatment of Merkel cell carcinoma and urothelial carcinoma. Anti-PD-L1 and anti-PD-1 MAbs are fairly well tolerated with a unique adverse event (AE) profile that includes an increased risk for development of immune-related AEs (irAEs) [6, 7].

Determinants of response to immune checkpoint inhibition include tumor mutational burden, expression of PD-L1 and PD-L2 in tumor cells, and the tumor microenvironment [8–10]. TETs in general, and thymomas in particular, have a low frequency of somatic mutations [11, 12]. However, thymic epithelial cells are known to express PD-L1 with the frequency of expression ranging from 23 to 68% in thymomas and 70 to 75% among thymic carcinomas [13–15].

Due to the high frequency of expression of PD-L1 in TETs, we decided to evaluate the safety and clinical activity of PD-L1 inhibition employing avelumab in patients with relapsed thymoma.

### Patients and methods

#### Study oversight

All patients provided written informed consent for participation in a clinical trial that was approved by the Institutional Review Board at the National Cancer Institute (NCT01772004) [16].

#### Study procedures

Patients with advanced solid tumors treated with at least one prior standard therapy were enrolled on a phase I, dose-escalation trial of avelumab (MSB0010718C) [17]. Avelumab was obtained via a Cooperative Research and Development Agreement (CRADA) between the National Cancer Institute (NCI) and EMD Serono. Key eligibility criteria included no prior use of immune checkpoint inhibitors (ICIs) and absence of autoimmune disease. All patients received avelumab intravenously over 60 min once every 2 weeks. NCI Common Terminology Criteria for Adverse Events (CTCAE) version 4.0 was used for toxicity assessment. Response Evaluation Criteria in Solid Tumors (RECIST), version 1.1 was used for tumor response evaluation performed every 6 weeks.

#### Multiplex immunohistochemistry

Formalin-fixed, paraffin-embedded (FFPE) tumor tissue sections were evaluated by multiplex immunohistochemistry (IHC) for tissue morphology, distribution of various immune cell subsets and antigen presentation markers. Immunoprofiling of tumor sections was performed using a novel high order immunofluorescence multiplexing platform [18], Cell-DIVE™ (GE Healthcare). This platform allowed single cell characterization of over 60 markers in a single FFPE tissue section, allowing spatial profiling of immune and other cell types as well as cellular states.

Targets used for identification included: CD3, CD4, CD8 and CD45RO for T cells (helper, cytotoxic and memory T cells); CD3, CD4, and FoxP3 for regulatory T cells (Tregs); CD3, CD20 and CD79 for B and plasma cells; CD3 (negativity) and CD16 for NK cells; and CD68 and CD163 for myeloid cells/macrophages. Also included were HLA-I, HLA-II and a host of other immune cell activation/inhibition markers. Only a subset of these are discussed here.

#### Evaluation of PD-1 and PD-L1 expression and tumor immune infiltrates

FFPE tumor tissue sections were evaluated by hematoxylin and eosin (H&E) staining. Immunostaining was performed for detecting expression of TdT, CD1a, CD4, CD3, CD8, CD5, PD-1 and PD-L1. Review of tumor specimens was performed by two pathologists (LB and SP).

#### Peripheral blood immune subset analyses

Eleven-color flow cytometry evaluations previously described [19, 20] were performed before and after administration of avelumab for the patients described in this paper and 28 patients with other cancers. The frequency of 123 peripheral blood mononuclear cell (PBMC) subsets using 30 immune cell markers was evaluated (Additional file 1: Table S1; online only).

Unsupervised hierarchical clustering of peripheral immune subsets prior to therapy with avelumab was performed in RStudio; the resulting heatmap was also generated using the same software. For the heatmap, raw data included major subsets (> 0.05% of PBMC) as a percent of total PBMC. Subsets were log 2 transformed and scaled to compute a normalized z-score for each attribute, and samples were clustered with the Complete Method.

#### T-cell receptor (TCR) sequencing analysis

DNA was extracted from cryopreserved PBMC before and after therapy using the Qiagen DNeasy Blood and Tissue Kit (Qiagen). TCR Vβ CDR3 sequencing (TCRseq) was performed at the NCI Genomics Core facility (Frederick, MD) using the deep resolution immunoSEQ platform (Adaptive Biotechnologies); analysis was performed using the immunoSEQ Analyzer 3.0 (Adaptive Biotechnologies). Repertoire size, a measure of TCR diversity, was determined by calculating the number of individual clonotypes represented in the top 25th percentile by ranked molecule count after sorting by abundance; this measure is relatively stable to differences in sequencing depth, and not strongly influenced by rare clonotypes.

## Results

### Retrospective analysis of PD-L1 expression in TET samples

In preparation for this study, we sought to determine PD-L1 expression in archived TET samples by IHC. Resection or biopsy specimens were obtained from 54 patients with the following clinical characteristics: median age 47 years (range, 17–77); 35 males/19 females; World Health Organization histology: Thymoma = 19 (AB 1, B1 1, B2 8, B3 8, unclassified thymoma 1), Thymic carcinoma = 35; Masaoka stage: IIB 1, III 1, IVA 16, IVB 36; history of prior systemic therapy in 45 (83%) cases.

PD-L1 expression was observed in 16 (84%) thymomas and 17 (49%) thymic carcinomas, whereas it was focal in 14 (40%) thymic carcinomas, and absent in three (16%) thymomas and four (11%) thymic carcinomas. Representative images of PD-L1 expression are shown in Additional file 2: Figure S1; online only.

### Patient characteristics

Seven patients with recurrent thymoma and one patient with recurrent thymic carcinoma were enrolled and received avelumab at doses of 10 mg/kg to 20 mg/kg intravenously every 2 weeks. Patient characteristics are presented in Table 1.

**Table 1:** Patient Characteristics

Patient Characteristics	N (%)
Age (years), Median (range)	53 (39–76)
Sex	
Male	5 (63)
Female	3 (37)
Race	
White	7 (88)
Black	1 (12)
Histology	
Thymoma	7 (88)
B1	1
B2	3
B3	3
Thymic carcinoma	1 (12)
Stage at presentation	
IVA	1 (12)
IVB	7 (88)
Previous treatment	
Systemic therapy^a^, Median (range)	3.5 (2–10)
Thymectomy^b^	7 (88)
Chest radiation therapy	7 (88)

^a^ Includes: Cisplatin + Doxorubicin + Cyclophosphamide (PAC), PAC + Prednisone, PAC + Belinostat, Cisplatin + Etoposide, Carboplatin + Etoposide, Carboplatin + Paclitaxel, Gemcitabine + Capecitabine, Cisplatin, Paclitaxel, Gemcitabine, Pemetrexed, Sunitinib, Cixutumumab, Milciclib, Octreotide, Amrubicin, Saracatinib, and Belinostat. ^b^Includes one case of debulking surgery

### Anti-tumor response

Overall, an objective response was observed in four of seven (57%) patients with thymoma, and the response could be confirmed with repeat imaging in two of seven (29%) patients. Details and duration of response are summarized in Table 2.

**Table 2:** Clinical Activity of Avelumab in Thymic Epithelial Tumors

Patient Number	WHO Histology	Treatment Dose (mg/kg)	Best Response	Number of Doses	Duration of Response (weeks)
1	B3 thymoma	20	cPR	1	14
2	B3 thymoma	20	SD	3	–
3	B2 thymoma	20	uPR	1	17
4	Thymic carcinoma	10	SD	8	–
5	B2 thymoma	10	SD	18	–
6	B2 thymoma	10	uPR	1	4
7	B3 thymoma	10	PD	6	–
8	B1 thymoma	10	cPR	10	12

*cPR* confirmed partial response, *SD* stable disease, *uPR* unconfirmed partial response, *PD* progressive disease

Three patients were treated with avelumab at the 20 mg/kg dose level. One patient had a confirmed partial response (WHO B3 thymoma, maximum tumor change from baseline: 48% after one dose of avelumab), one patient had an unconfirmed partial response (WHO B2 thymoma, maximum tumor change: 30% after one dose of avelumab) and one patient had stable disease (WHO B3 thymoma, maximum tumor change: 8% reduction after three doses of avelumab).

Four patients with thymoma (WHO B1 = 1; WHO B2 = 2; WHO B3 = 1) and one patient with thymic carcinoma received avelumab at a dose of 10 mg/kg. The patient with B1 thymoma had a confirmed partial response and one patient with a B2 thymoma had an unconfirmed partial response with maximum tumor shrinkage of 37 and 31%, respectively. One patient with B2 thymoma and the patient with thymic carcinoma had stable disease with no tumor shrinkage and the patient with B3 thymoma had disease progression.

**Fig. 1:**
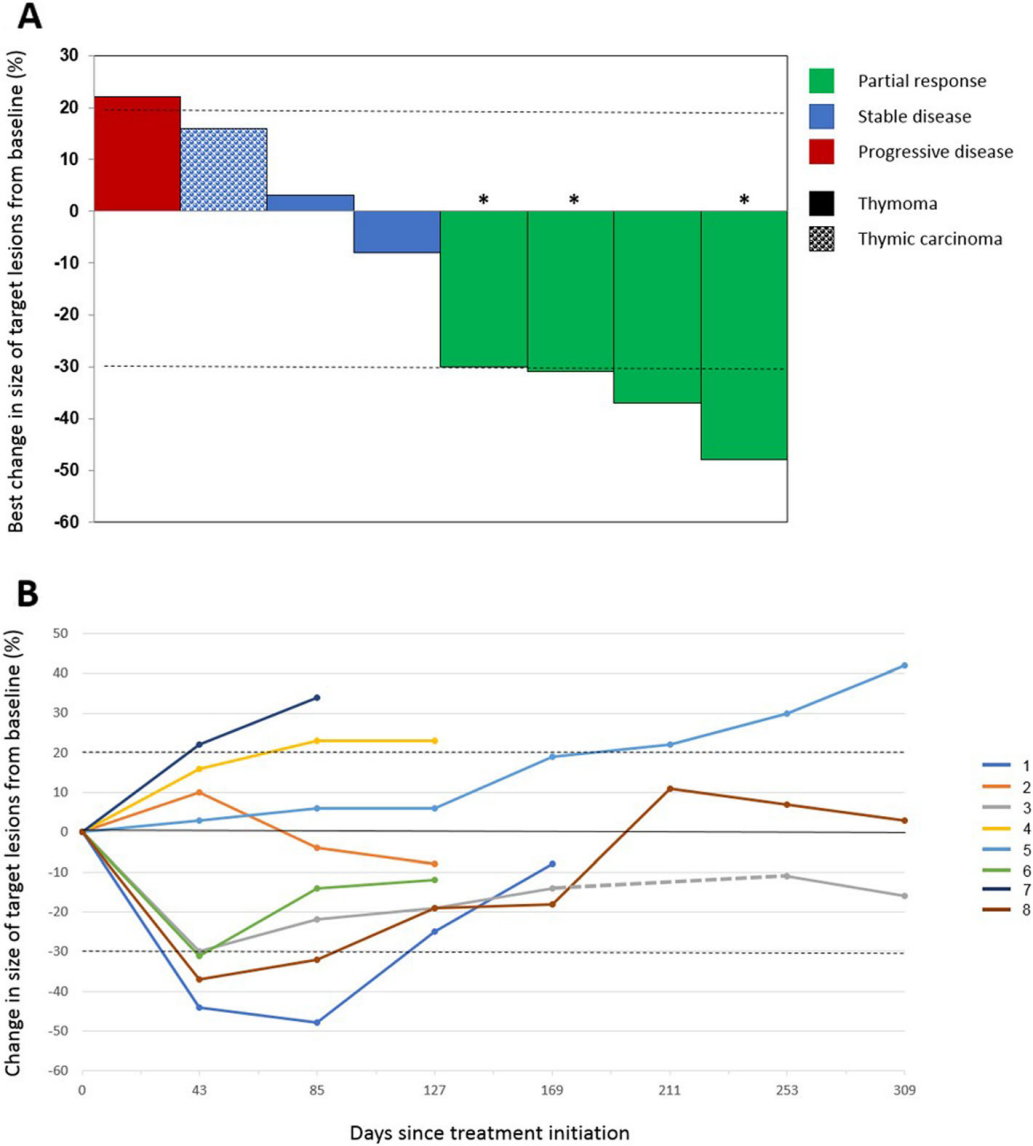
Response to therapy and duration of response. **a** Waterfall plot of best response to treatment. Four patients with thymoma achieved a partial response to treatment, including three patients who received only one dose of avelumab (*). Patients with any tumor shrinkage also developed irAE. **b** Duration of response. Change in the size of target lesions over time during treatment and after discontinuation of therapy (until the last follow-up time point) is illustrated. Three patients (1, 3, and 6) received one dose of avelumab

**Fig. 2:**
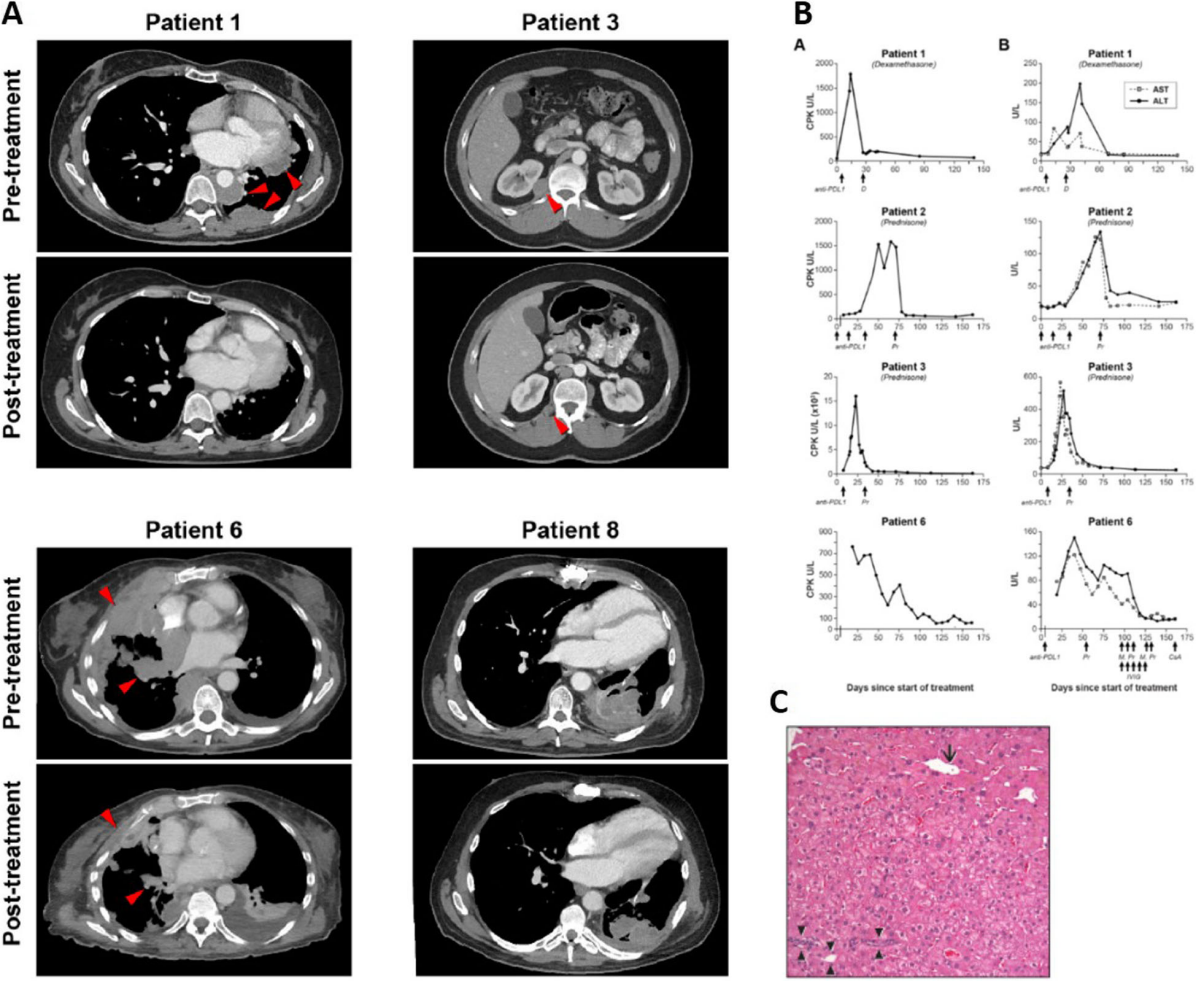
Changes in radiographic appearance of tumor and laboratory parameters after treatment. **a** Changes in selected target lesions in patients responding to treatment. Representative axial CT images of patients achieving a partial response to treatment showing the maximum change in size of selected tumor lesions. **b** Biochemical changes in response to treatment with avelumab. Column A, on the left, shows changes in CPK. Column B, on the right, shows changes in AST and ALT. Three out of four patients (1, 3, and 6) also developed a radiological response to treatment. In these three cases, only one dose of avelumab could be administered due to the development of autoimmunity. Days of administration of avelumab and other medications are indicated by arrows. **c** Post-treatment liver core biopsy from patient 1 with portal space (arrowheads), centrilobular vein (arrow) and no evidence of inflammation. CPK: creatinine phosphokinase, AST: aspartate transaminase, ALT: alanine transaminase, D: dexamethasone, Pr: prednisone, M.Pr: methylprednisolone, IVIG: intravenous immunoglobulin, CsA: cyclosporine A

Response to therapy and duration of response are illustrated in Fig. 1. Figure 2a illustrates changes in selected target lesions in patients responding to treatment.

### Toxicity

Treatment-related AEs are summarized in Table 3. Grade 3 and 4 AEs were observed in 3 (38%) patients each. Most AEs were mild (grade 1 or 2) and consistent with previously observed toxicities associated with ICIs. However, a disproportionately large number of patients developed signs and symptoms suggestive of autoimmunity that were included under the umbrella term “autoimmune disorder,” which accounted for all the grade 3 and 4 AEs observed in this series (with the exception of one case of grade 4 hyperkalemia in a patient with grade 3 diarrhea due to autoimmune enteritis). These AEs included muscle weakness, myalgia, myositis, respiratory muscle insufficiency, hoarseness, paresthesia, dysphagia, dyspnea, diarrhea and elevated creatine phosphokinase (CPK). Details of irAEs are presented below and in Additional file 1: Table S2; online only. Neuromuscular AEs observed in our series have been reported separately [21].

**Table 3:** Adverse Events, at Least Possibly Related to Treatment with Avelumab

Adverse Event	Grade 1	Grade 2	Grade 3	Grade 4
Tumor pain	1 (13%)			
Back pain		1 (13%)		
Extremity pain	1 (13%)			
Fever	1 (13%)			
Flu-like symptoms	1 (13%)			
Chills	1 (13%)			
Fatigue	3 (38%)	1 (13%)		
Nausea	1 (13%)			
Wheezing	1 (13%)			
Bronchial infection		1 (13%)		
Ear and labyrinth disorder (fullness)		1 (13%)		
Urinary urgency		1 (13%)		
Autoimmune disorder			3 (38%)	2 (25%)
Hypokalemia				1 (13%)
Hypomagnesemia		1 (13%)		

Patient 1 (stage IVA, WHO subtype B3 thymoma) developed grade 3 CPK elevation and grade 1 transaminitis 2 weeks after administration of avelumab (Fig. 2b). Oral steroids were started on day 18 and tapered over a 6-week period with resolution of laboratory abnormalities. A liver biopsy performed on day 43 showed no evidence of drug-induced liver injury despite elevated transaminases (Fig. 2c). The patient was not re-challenged with avelumab.

Patient 2 (stage IVB, WHO B3 thymoma) received three doses of avelumab uneventfully before an elevation in CPK and liver transaminases was noted (Fig. 2b). The patient developed bulbar weakness with mild sensory loss in feet, facial diplegia, weakness of the tongue and hypophonia. Avelumab was discontinued; oral steroids were started on day 49 and discontinued on day 171 with partial resolution of symptoms.

Patient 3 (stage IVB, WHO subtype B2 thymoma) experienced an increase in CPK 1 week after starting avelumab and it peaked 23 days later. Magnetic resonance imaging (MRI) of bilateral thighs showed changes consistent with myositis. Changes in laboratory parameters are shown in Fig. 2b. Oral steroids were initiated on day 23 and laboratory abnormalities resolved gradually. Steroids were discontinued on day 87. The patient was not re-treated with avelumab.

Patient 6 (stage IVA, WHO B2 thymoma) developed grade 2 dysphagia and generalized muscle weakness 2 days after starting avelumab. CPK, aspartate transaminase (AST) and alanine transaminase (ALT) elevation were observed 8 days after treatment and oral prednisone was started at a dose of 60 mg per day (Fig. 2b). Due to worsening dyspnea and dysphagia and a vital capacity of 790 ml, the patient was admitted to the intensive care unit 13 days after treatment and underwent elective intubation and mechanical ventilation. After three doses of intravenous methylprednisolone and five doses of intravenous immunoglobulin, a transient decrease in CPK, AST and ALT was observed. Two more doses of methylprednisolone were administered on days 18 and 19, resulting in normalization of CPK. However, due to persistently elevated transaminases and the need for continued ventilatory support, intravenous cyclosporine A was started on day 23, resulting in gradual resolution of transaminitis within 3 weeks. Recovery from respiratory failure was partial and prolonged.

Patient 8 (stage IVB, WHO B1 thymoma) received 11 doses of avelumab before developing grade 3 diarrhea. Colonoscopy and small bowel biopsy revealed ileitis with villous blunting and active inflammation (not shown). Diarrhea subsided after treatment with oral prednisone. Despite the presence of autoimmune enteritis, no abnormalities of CPK or liver transaminases were observed.

Six of eight patients had received sunitinib previously, including all four patients with an objective response to avelumab. These four responding patients also developed irAEs as described below. Of the remaining two patients previously treated with sunitinib who had stable disease with avelumab, one developed irAEs (only the patient with thymic carcinoma received prior sunitinib and did not develop irAEs). In two out of eight patients (both with B2 thymoma) who had not received prior sunitinib, one had stable disease and one had progressive disease with avelumab; neither developed irAEs. Details of a possible association of response and irAEs with prior sunitinib are presented in Additional file 1: Table S3; online only.

Interestingly, development of a response was accompanied by irAEs (myositis in three cases and enteritis in one case). Patients with clinical evidence of myositis developed sustained elevations of CPK, AST and ALT as illustrated in Fig. 2b. Post-treatment tissue inflammation was also demonstrated by imaging studies (MRI of the thighs showed myositis, and cardiac MRI showed myocarditis), histopathological analysis (small bowel biopsy in the patient developing enteritis) and electrophysiological studies (myopathic findings on an electromyogram in two of three patients with myositis).

The temporal association between onset of steroid therapy and evidence of tumor shrinkage is depicted in Additional file 1: Table S4; online only. Tumor shrinkage was observed either before development of irAEs and use of steroids (in one case) or shortly after onset of steroid therapy (9 and 20 days later in two cases). This observation suggests that tumor response was related to avelumab rather than steroids used to treat irAEs.

## Tissue and blood immunologic analyses

### Tumor PD-1 and PD-L1 expression

Paired tumor biopsies were analyzed for PD-L1 expression in three patients. In one of three cases the post-treatment biopsy showed necrotic tissue with no viable tumor and was not suitable for immunohistochemical analysis. In the other two cases, diffuse membranous staining pattern in the epithelial component was seen in both pre- and post-treatment biopsies (Additional file 3: Figure S2; online only). Additionally, scattered PD-1 positive lymphocytes were seen before and after treatment (Additional file 3: Figure S2; online only). Staining of normal thymus for control purposes also showed scattered PD-1-positive lymphocytes, predominantly in the medulla (not shown).

### Analysis of tumor immune infiltrates

Intratumoral immune infiltrates before and after treatment were also evaluated in the two cases described above (Additional file 3: Figure S2; online only). The immune infiltrate in pre-treatment tumor samples of both patients was composed of immature T cells expressing TdT, CD1a and CD5, CD4 and CD8. However, the lymphoid infiltrate in the post-treatment biopsy in one case did not express TdT or CD1a and showed primarily lymphocytes with a mature CD8 positive T-cell phenotype. This patient had a confirmed partial response to treatment. In contrast, the immune infiltrate in the post-treatment biopsy for the other patient showed a phenotype consistent with immature T cells (thymocytes) expressing TdT, CD1a and both CD4 and CD8. This patient had stable disease in response to treatment.

### Multiplex immunoprofiling of tumor samples

#### Innate and adaptive immune cells

**Fig. 3:**
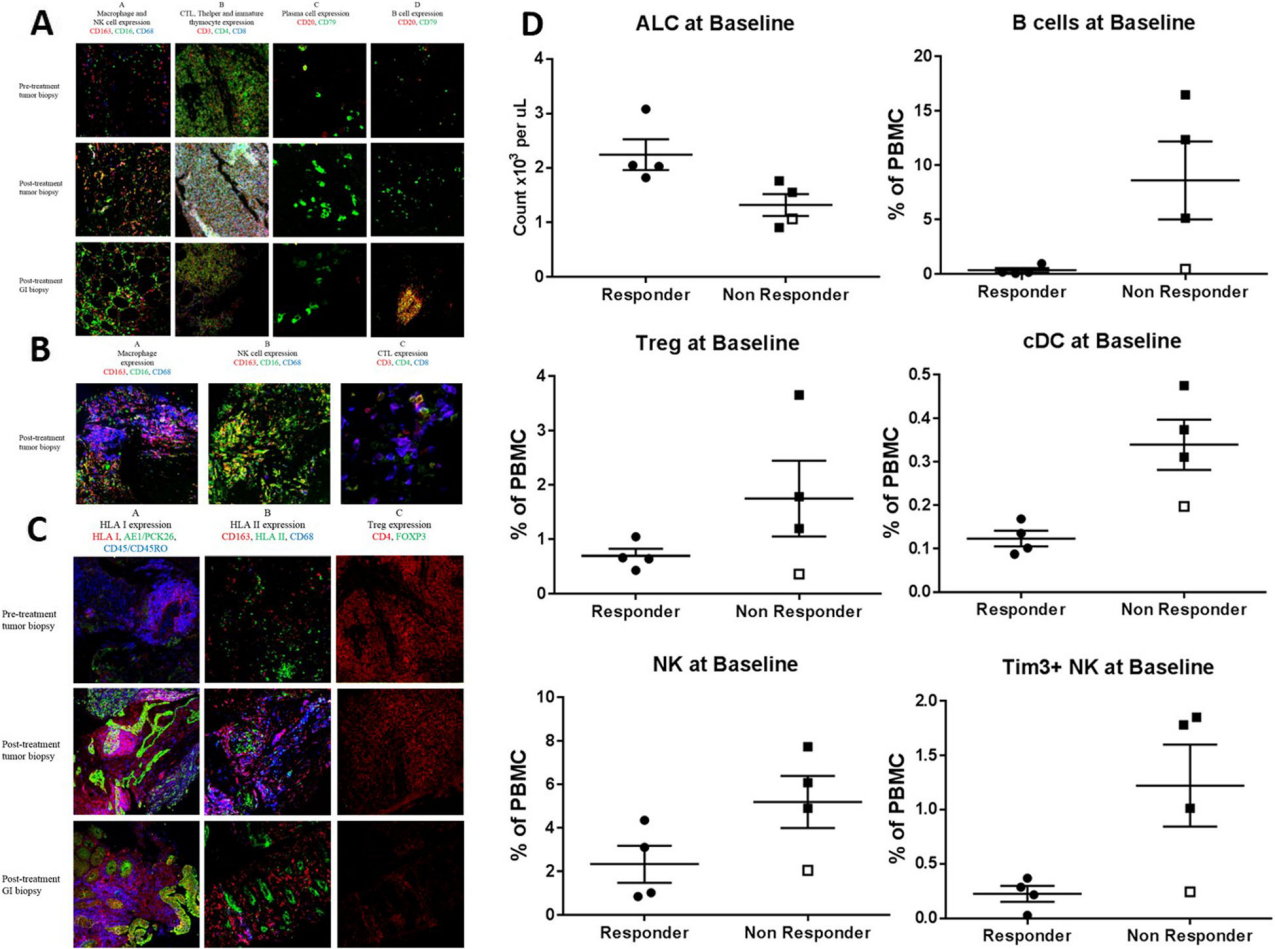
Tumor and blood immunologic analyses. **a** Pre-treatment tumor biopsy and post-treatment tumor and gastrointestinal (GI) biopsies from patient 8 showing expression of macrophages in different stages of differentiation (shown in red, purple and orange due to expression of different combinations of markers), and natural killer (NK) cells (green) in column A, cytotoxic T lymphocytes (CTL; blue/purple), helper T cells (Thelper; green/orange) and immature thymocytes (white) in column B, plasma cells (green) in column C, and B cells (red/orange) in column D. Higher macrophage, NK cell and CTL expression was seen in tumor samples after treatment. Scattered plasma cells were observed with no appreciable change after treatment. No significant B cell population was observed, except in one field of view of the GI biopsy (shown above). **b** Post-treatment tumor biopsy from a lesion demonstrating response from patient 3 showing expression of macrophages (red, blue, purple and orange) in Panel A, natural killer (NK) cells (green) in Panel B, and cytotoxic T lymphocytes (CTL, blue/purple) in Panel C. **c** Pre-treatment tumor biopsy and post-treatment tumor and gastrointestinal (GI) biopsies from patient 8 after nine doses of avelumab showing increased expression of HLA I (red/pink/purple due to overlap with pan-leukocyte marker in blue, or orange due to overlap with pan-cytokeratin marker in green; column A), low and heterogeneous expression of HLA II (green) surrounded by macrophages (red/blue/purple) (column B) in post-treatment samples. Regulatory T cells (Tregs) were not present in significant numbers in pre- and post-treatment samples (column C). **d** Absolute lymphocyte count (ALC) and frequency of immune cell subsets prior to therapy (baseline) that are differentially expressed between clinical responders (patients 1, 3, 6, and 8) and non-responders (patients 2, 4, 5, 7). Patient 2 (a clinical non-responder who developed autoimmune adverse event like the responders) is noted with an open square. cDC, conventional dendritic cells

Pre-treatment tumor biopsy and post-treatment tumor and gastrointestinal tract biopsies were analyzed for patient 8, who achieved a partial response to treatment but developed enteritis. Higher macrophage (in various stages of differentiation), natural killer (NK) cell and cytotoxic T lymphocyte expression was observed after treatment (Fig. 3a). Biopsies obtained from the gastrointestinal tract upon development of enteritis also showed high macrophage, NK cell and cytotoxic T lymphocyte expression, although no pre-treatment intestinal biopsies were available for comparison (Fig. 3a). Both pre- and post-treatment biopsies revealed scattered plasma cells and no significant population of B cells (Fig. 3a).

Patient 3 achieved a partial response after one dose of avelumab. A paravertebral soft tissue mass that responded to treatment was biopsied and showed widespread necrosis, no viable tumor and expression of macrophages, NK cells and cytotoxic T lymphocytes (Fig. 3b).

#### Human leukocyte antigen and regulatory T-cell expression

Increased expression of HLA I was observed in post-treatment tumor and gastrointestinal (GI) tract biopsies from patient 8 after nine doses of avelumab (Fig. 3c). HLA II expression was low and heterogeneous and observed in pre- and post-treatment tumor biopsies. HLA II expression was also observed in gastrointestinal epithelial cells. Tregs were not present in significant amounts in pre- and post-treatment tumor biopsies (Fig. 3c).

### Analyses of peripheral blood immune cell subsets

PBMC were monitored at various times pre– and post–anti-PD-L1 therapy for 123 immune cell subsets. The most profound differences were observed prior to therapy between patients who did or did not develop a clinical response to avelumab. Compared to non-responders, responders had a higher absolute lymphocyte count, and lower frequencies of B cells, Tregs, conventional dendritic cells (cDCs), and NK cells before treatment (Fig. 3d). While some of these differences were statistically significant, they should only be considered trends, due to the small number of patients analyzed. Patient 2, a non-responder who developed irAEs like the responders, had an immune profile more similar at baseline to responders than non-responders, including very low levels of Tregs and B cells. Decreases in Tregs and increases in myeloid derived suppressor cells (MDSC) were also noted following steroids in clinical responders who developed irAEs (Additional file 4: Figure S3; online only).

**Fig. 4:**
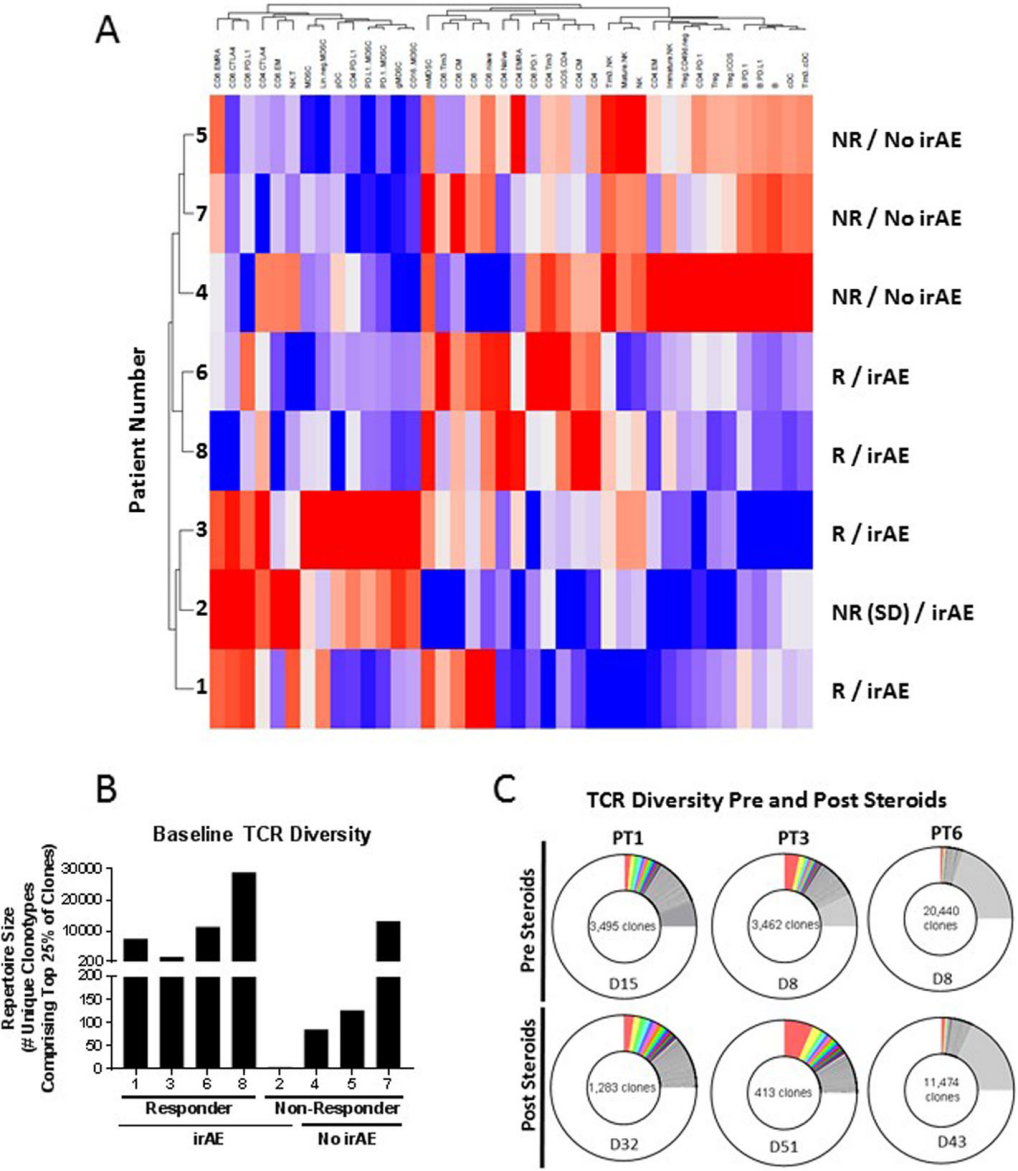
Immune phenotype associated with development of clinical responses and autoimmunity, and the effect of steroids. **a** Unsupervised hierarchical clustering of indicated immune populations in PBMC prior to treatment with avelumab. Higher levels of expression are indicated in red and lower levels of expression are indicated in blue. Patient response (R, responders; NR, non-responders) and development of immune-related adverse event (irAE) are indicated. **b** Diversity of TCR repertoire in PBMC of patients prior to therapy with avelumab. **c** Diversity of the TCR repertoire in PBMC of patients pre- and post-steroid treatment. TCR diversity was measured by the metric of repertoire size; values in panels B and C indicate the number of individual clonotypes comprising the top 25th percentile by ranked molecule count after sorting by abundance. The day (D) PBMC were assessed for TCR diversity pre- and post-steroids is indicated

Unsupervised hierarchical clustering of major PBMC subsets prior to therapy was employed to determine if any signature emerged that would distinguish clinical responders (R) vs. non-responders (NR). As seen in Fig. 4a, the five patients who developed irAEs (four of whom also had a radiological response) clustered separately, thus confirming and expanding on the results shown in Fig. 3d, which was performed employing retrospective clinical data.

TCR diversity in PBMC prior to therapy was also analyzed. As seen in Fig. 4b, there was a trend towards a higher level of TCR diversity in those patients who subsequently had a radiological response and developed irAEs. PBMC were available from three patients pre- and post-steroid administration. As seen in Fig. 4c, there was a clear decrease in TCR diversity in all three patients post-steroids.

## Discussion

We report major tumor regressions in four of seven (57%) patients with recurrent thymoma treated with the anti-PD-L1 antibody, avelumab. Response was associated with development of irAEs in these patients with no preexisting history of thymoma-associated autoimmune disease. Among non-responders, only one of three patients experienced irAEs. Three of four responders could receive only one dose of avelumab due to the development of AEs. Despite this, significant tumor responses were observed, and no RECIST-defined disease progression was seen for 14 weeks or more in two of three cases. irAEs were medically manageable, demonstrated a unique pattern (high frequency of myositis, myocarditis and neuromuscular AEs) in patients with thymoma, and have been reported previously in response to PD-1 inhibition in TET patients [22, 23]. A similar pattern and frequency of irAEs has not been reported in patients with other solid tumors treated with avelumab, or other anti-PD-1/anti-PD-L1 antibodies [17, 24–27].

These data suggest that patients with TETs, particularly thymoma, are predisposed towards the development of musculoskeletal, neuromuscular and cardiac irAEs in response to immune checkpoint inhibition for as yet unclear reasons. It should be noted that unsupervised hierarchical clustering of PBMC analyses prior to therapy by flow cytometry revealed a dichotomy in phenotype of those patients who subsequently responded to therapy and developed irAEs. Moreover, there was a trend in those same patients towards having a higher level of TCR diversity in PBMC prior to therapy. TCR diversity in PBMC also decreased in patients treated with steroids. It should be noted that patient 6 had an extremely high level of TCR diversity prior to steroids; this was the same patient who developed the most severe irAEs.

The anti-tumor effect seen in our patients could be related to the known mechanism of action of anti-PD-L1 MAbs, i.e., blockade of the binding of PD-L1 to PD-1 activates antigen-specific T cells that destroy tumor cells bearing target antigens [10]. However, anti-tumor activity could also be due to a direct effect of avelumab via antibody-dependent cell-mediated cytotoxicity, since it is a fully human IgG1 MAb [28]. In one of two patients with post-treatment tumor tissue available for analysis, replacement of thymocytes with mature CD8 positive T cells was observed.

AEs in our patients could be attributable to the induction of autoimmunity because of a biological predisposition arising from the underlying thymoma. It is known that thymic epithelium exhibits “promiscuous gene expression” for the process of negative selection, suggesting that a tightly controlled immunomodulatory system is destabilized as a result of PD-L1 blockade in patients with thymoma [29]. PD-L1 expression has also been detected in thymic epithelial and stromal cells, especially in lymphocyte-rich thymomas [13–15]. We hypothesize that under these conditions blockade of the PD-1/PD-L1 pathway results in the disinhibition of effector T cells that are capable of inducing thymic epithelial cell death and overcoming immunological tolerance against normal tissue antigens expressed on thymic epithelium [29, 30].

Interestingly, all patients with advanced thymoma responding to avelumab had received sunitinib previously. Moreover, all patients who developed avelumab-related irAEs were also treated with sunitinib previously. Two of three patients who did not develop irAEs had not been exposed to sunitinib (the third patient without avelumab-related irAEs had thymic carcinoma). Although these numbers are small, our observations raise the possibility of previous sunitinib exposure influencing the development of response to ICI therapy and increasing the risk of irAEs in patients with thymoma. Sunitinib is a multikinase inhibitor with activity in advanced thymic carcinoma [31]. It has well-described immunomodulatory properties and has been shown to decrease the population of Tregs and MDSCs at therapeutic doses [32–34]. These effects can potentially explain tumor response and irAEs seen in our patients as described below.

Tregs are known to help in the maintenance of immunological tolerance and a reduction in Tregs favors the development of autoimmune disease [35]. Our observations of a lower level of Tregs before treatment in responders who developed irAEs compared to non-responders may support the clinical observations of the generation of an anti-tumor response accompanied by the development of irAEs. Whether these observations can be explained exclusively by changes in T-cell activity or whether a B-cell–related, antibody-dependent process targeting normal human tissue is also involved is yet to be determined. It is conceivable that treatment with immunomodulatory drugs like sunitinib can prime the immune system and increase the likelihood of response and increase the risk of toxicity related to ICIs in patients with advanced TETs.

An increased predisposition towards development of irAEs in response to immune checkpoint inhibition in patients with thymoma makes it necessary to develop strategies to identify patients at high risk before initiation of treatment. Conventionally, patients with a history of autoimmune disease are not offered treatment with immune checkpoint inhibitors and were excluded from our clinical trial as well. We have published a separate report on an association between the development of myositis observed in our trial and the presence of B cell cytopenia and muscle acetylcholine receptor autoantibodies prior to treatment [21]. If validated in future studies, these parameters might serve as markers of pre-existing autoimmunity in patients without a clinical history of autoimmune disease and identify individuals at high risk of myositis and other irAEs. These markers and other risk mitigation strategies are under evaluation in an ongoing trial of avelumab in patients with advanced TETs (NCT03076554) [36].

Finally, despite the unique aspects of TET biology, a few observations similar to ours have been described in non-thymic cancers such as a broadening of the TCR repertoire at 2 weeks post-initiation of treatment and preceding irAE onset in patients with metastatic prostate cancer receiving anti-CTLA4 and anti-PD-1 therapy [37], a decline in circulating B cells in response to immune checkpoint blockade in melanoma patients developing high-grade irAEs [38], and a greater likelihood of melanoma patients achieving disease control after treatment with ipilimumab if they had higher absolute lymphocyte and lower Treg counts at baseline [39]. These findings suggest that certain mechanisms of response and toxicity related to immune checkpoint inhibition transcend the biology of the underlying tumor.

Our observations provide a rationale to evaluate a broader range of variables in other cancers as potential biomarkers of response (pretreatment B cell, cDC and NK cell counts) and immune-related toxicity (pretreatment B cell count).

## Conclusions

In conclusion, we observed promising antitumor activity, which suggests that further clinical investigation of anti-PD-L1 therapy in patients with recurrent thymoma is warranted. Response is associated with an increased propensity to develop an unusual pattern of irAEs. However, we also demonstrated that most AEs can be managed with systemic steroids. A better understanding of the nature of autoimmune toxicity and its management is needed to ensure the safety and feasibility of using ICIs in patients with thymoma.

## Additional files

Additional file 1: **Table S1.** Flow-cytometry analysis of immune subsets. Subsets analyzed included 9 standard immune subsets (PD-L1 and PD-1 expression analysis was performed for all standard subsets) and 96 subsets relating to maturation and function of immune cells. **Table S2.** Details of autoimmune adverse events associated with treatment. **Table S3.** Association of avelumab-related response and irAEs with prior treatment with sunitinib. **Table S4.** Association between steroid use and response in patients responding to treatment. (DOCX 96 kb)

Additional file 2: **Figure S1.** PD-L1 expression in thymoma and thymic carcinoma. **(A, E)** WHO B3 thymoma with 95% thymic epithelial cell PD-L1 positivity and 3+ intensity. **(B, F)** WHO B2 thymoma with 50% thymic epithelial cell PD-L1 positivity and 2+ intensity. **(C, G)** Thymic carcinoma with 90% thymic epithelial cell PD-L1 positivity and 2+ intensity. **(D, H)** Thymic neuroendocrine carcinoma with focal thymic epithelial cell PD-L1 positivity and 1+ intensity. (**A-D**: 10x magnification; **E-H**: 40x magnification.). (JPG 123 kb)

Additional file 3: **Figure S2**. Pre- and post-treatment immunohistochemical evaluation of PD-1, PD-L1, CD4, CD8, TdT and CD1a for patients 1 and 2. PD-1 staining showed scattered PD-1–positive lymphocytes and PD-L1 staining showed diffuse membranous pattern in the epithelial component of the thymoma in both patients. In tissue sections obtained from patient 1 (A), a pre-treatment pleural lesion showed sheets of epithelial cells with abundant well-defined cytoplasm and oval nuclei with a fine chromatin pattern and sparse lymphocytes. Lymphocytes within the tumor expressed CD4, CD8, TdT and CD1a, a pattern consistent with thymocytes. A post-treatment biopsy of a peri-hepatic mass showed morphological characteristics similar to those in the pre-treatment pleural lesion biopsy. However, lymphocytes within the peri-hepatic mass did not express TdT or CD1a; a few CD4 positive cells were seen but the majority of lymphocytes showed only CD8 expression. In tissue sections obtained pre- and post-treatment from patient 2 (B), epithelial cells were seen with abundant well-defined cytoplasm and interspersed lymphocytes. The lymphocytic component in both specimens showed a similar phenotype expressing CD4, CD8, TdT and CD1a consistent with thymocytes. (JPG 82 kb)

Additional file 4: **Figure S3.** Decrease in regulatory T cells (Tregs) (A-D) and increase in myeloid derived suppressor cells (MDSC) (E-H) following steroids in clinical responders who developed immune-related adverse events (irAEs). Patient 1 **(A, E)**, Patient 3 **(B, F**), and Patient 6 **(C, G)** received steroids for irAEs, while patient 8 **(D, H)** developed clinical response but no irAE for 60 days after documentation of response. Steroids were not used during this time frame. Dashed line denotes timing of steroids and solid line indicates time of clinical response. (JPG 74 kb)

## Data Availability

The datasets used and/or analyzed during the current study are available from the corresponding author on reasonable request.

## Abbreviations

AE: Adverse event
ALC: Absolute lymphocyte count
ALT: Alanine transaminase
AST: Aspartate transaminase
cDCs: Conventional dendritic cells
CPK: Creatine phosphokinase
CRADA: Cooperative Research and Development Agreement
CsA: Cyclosporine A
CTCAE: Common Terminology Criteria for Adverse Events
CTL: Cytotoxic T lymphocytes
FFPE: Formalin-fixed, paraffin-embedded
GI: Gastrointestinal
H&E: Hematoxylin and eosin
ICI: Immune checkpoint inhibitors
IHC: Immunohistochemistry
irAE: Immune-related adverse event
MAb: Monoclonal antibody
MDSC: Myeloid derived suppressor cells
MRI: Magnetic resonance imaging
NCI: National Cancer Institute
NK: Natural killer
PBMC: Peripheral blood mononuclear cells
PD-1: Programmed death-1
PD-L1: Programmed death-ligand 1
RECIST: Response Evaluation Criteria in Solid Tumors
TCR: T-cell receptor
TET: Thymic epithelial tumor
Tregs: Regulatory T cells
