# Supplementary material for: Efficacy and tolerability of anti-programmed death-ligand 1 (PD-L1) antibody (Avelumab) treatment in advanced thymoma
Source: J Immunother Cancer. 2019 Oct 21;7:269. doi: 10.1186/s40425-019-0723-9 (PMC6805423; doi:10.1186/s40425-019-0723-9)
Supplement: Supplementary file 1 — Table S1. Flow-cytometry analysis of immune subsets. Subsets analyzed included 9 standard immune subsets (PD-L1 and PD-1 expression analysis was performed for all standard subsets) and 96 subsets relating to maturation and function of immune cells. Table S2. Details of autoimmune adverse events associated with treatment. Table S3. Association of avelumab-related response and irAEs with prior treatment with sunitinib. Table S4. Association between steroid use and response in patients responding to treatment. (DOCX 96 kb) [file 40425_2019_723_MOESM1_ESM.docx]

**Table S1. Flow-cytometry analysis of immune subsets.** Subsets analyzed included 9 standard immune subsets (PD-L1 and PD-1 expression analysis was performed for all standard subsets) and 96 subsets relating to maturation and function of immune cells.


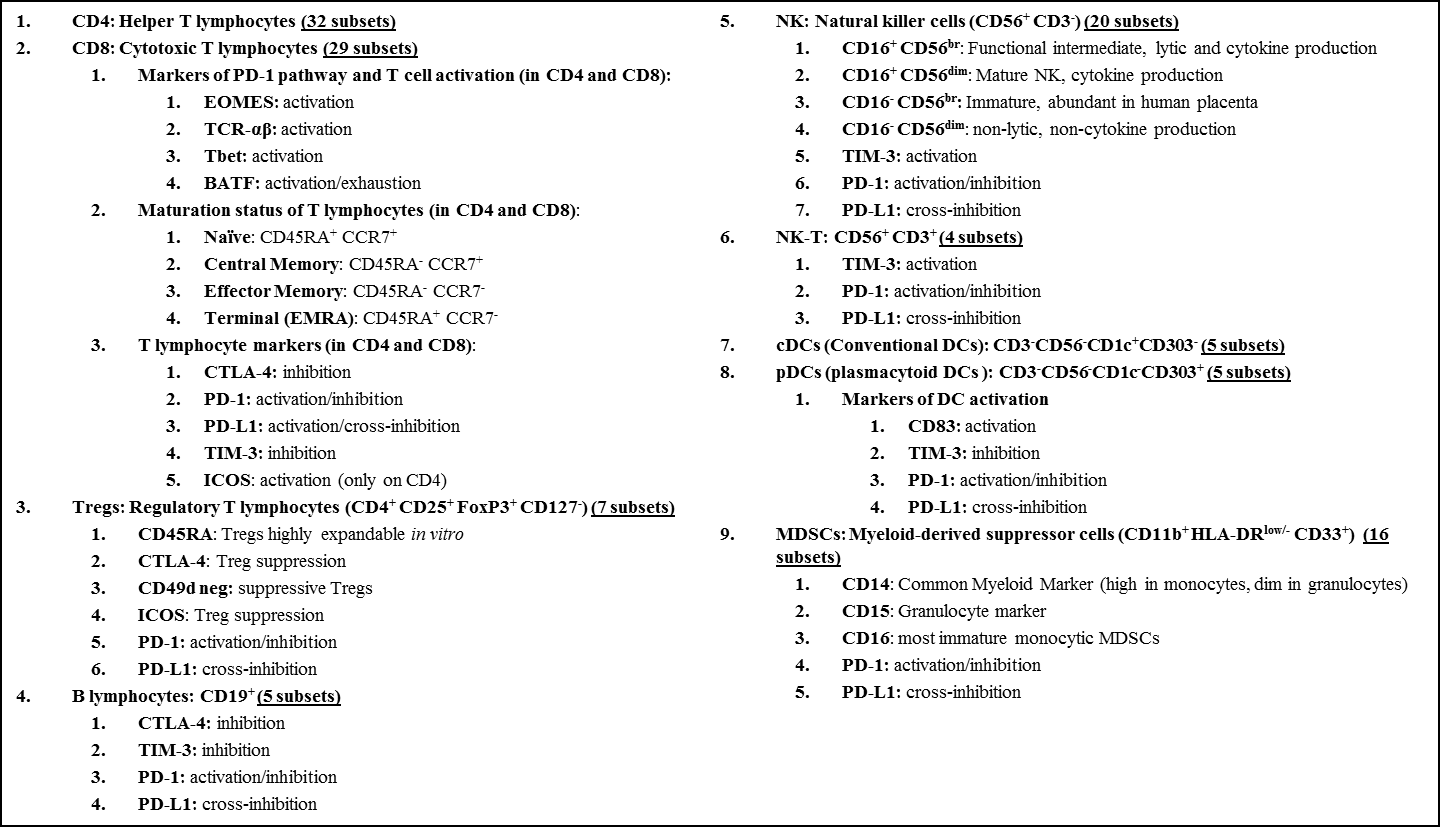


Derived from Donahue RN, ref 20.

**Table S2.** **Details of autoimmune adverse events associated with treatment.**

| **Adverse event** | **CTCAE grade** | | | | | **Comments** |
| --- | --- | --- | --- | --- | --- | --- |
|  | **Pt 1** | **Pt 2** | **Pt 3** | **Pt. 6** | **Pt. 8** |  |
| **Elevated CPK** | 4 | 3 | 4 | - | - | **Patient 1**: CPK elevation and myalgia observed 1 week after treatment. Peak CPK 3194 units/liter (ULN 234 units/liter). Dexamethasone started on day 18 with rapid normalization of CPK and complete resolution of symptoms.  **Patient 2**: CPK elevation noted 7 weeks after treatment. Peak CPK 1591 units/liter (ULN 308 units/liter). Prednisone started on day 49 with rapid normalization of CPK but incomplete resolution of symptoms. MRI of the thighs on day 50 showed no evidence of myositis.  **Patient 3**: CPK elevation observed 1 week after treatment. Peak CPK 16037 units/liter (ULN 308 units/liter). Myalgia on exertion started on day 18. Prednisone started on day 23 with rapid normalization of CPK and complete resolution of symptoms. MRI of the thighs on day 31 consistent with myositis. |
| **Elevated troponin** | 1 | 1 | 1 | - | - | **Patient 1**: Peak troponin I 0.67 ng/mL (ULN 0.045 ng/mL). No regional wall motion abnormalities on echocardiogram.  **Patient 2**: Asymptomatic. Peak troponin T 0.22 ng/ml (ULN 0.009 ng/mL).  **Patient 3**: Asymptomatic. Peak troponin T 0.99 ng/ml (ULN 0.009 ng/mL). |
| **Elevated liver transaminases**  **(AST and ALT)** | 2 | 2 | 3 | - | - | **Patient 1**: Asymptomatic. Peak AST 165 units/liter (ULN 40 units/liter); peak ALT 199 units/liter (ULN 41 units/liter). No evidence of hepatitis in liver biopsy.  **Patient 2**: Asymptomatic. Peak AST 126 units/liter; peak ALT 134 units/liter.  No serological evidence for autoimmune hepatitis and transaminitis resolved after initiation of steroids in all patients.  **Patient 3**: Asymptomatic. Peak AST 567 units/liter; peak ALT 376 units/liter. |
| **Shortness of breath** | 2 | 3 | - | 4 | - | **Patient 1**: Mild shortness of breath on exertion. Post-treatment PFTs not performed in the sitting and supine position. Gradual improvement in FVC observed with prednisone.  **Patient 2**: Asymptomatic. Post-treatment day 37 FVC 2.3 liters (47%) in the sitting position and 1.6 liters (33%) in the supine position.  **Patient 3**: Shortness of breath at rest. Post-treatment day 9 FVC 2.45 liters (47%) in the sitting position and 1.93 liters (37%) in the supine position. Gradual improvement in FVC observed with prednisone.  **Patient 6:** Shortness of breath within 1 week of avelumab treatment with rapid worsening and respiratory failure within 2 weeks. FVC was 790 milliliters on day 13 and the patient underwent elective intubation and mechanical ventilation for respiratory insufficiency. |
| **Cranial neuropathy** | - | 2 | - | - | - | **Patient 2**: Clinical and neurophysiological details will be presented in a separate manuscript. MRI of the brain was unremarkable. Flow cytometry of cerebrospinal fluid showed a predominant T cell population. Partial resolution with prednisone. |
| **Enteritis** | - | - | - | - | 3 | **Patient 8**: Colonoscopy showed small bowel enteritis. No response to budesonide but rapid and complete resolution with prednisone. |

CTCAE: Common Terminology Criteria for Adverse Events, version 4.0; CPK: creatine phosphokinase; FVC: forced vital capacity; MRI: magnetic resonance imaging; AST: aspartate transaminase; ALT: alanine transaminase; EMG: electromyogram; PFT: pulmonary function tests; ULN: upper limit of normal.

**Table S3. Association of avelumab-related response and irAEs with prior treatment with sunitinib.**

| **Patient no.** | **Start date of avelumab** | **Response to avelumab** | **Development of irAE** | **Prior treatment with sunitinib** | **Last dose of sunitinib relative to start of avelumab** |
| --- | --- | --- | --- | --- | --- |
| 1 | D1 | PR | Yes | Yes | D -77 |
| 2 | D1 | SD* | Yes | Yes | D -90 |
| 3 | D1 | PR^ | Yes | Yes | D -34 |
| 4 | D1 | SD | No | Yes | D -567 |
| 5 | D1 | SD | No | No | NA |
| 6 | D1 | PR^ | Yes | Yes | D -158 |
| 7 | D1 | PD | No | No | NA |
| 8 | D1 | PR | Yes | Yes | D -603 |

D: Day; irAE: immune-related adverse event; PR: partial response; SD: stable disease; *patient had a minor response (8%); ^unconfirmed partial response; NA: not applicable; PD: progressive disease.

**Table S4. Association between steroid use and response in patients responding to treatment.**

| **Patient no.** | **Treatment start date** | **Date of**  **irAE** | **Start of steroids** | **Evidence of tumor response** | **Discontinuation of steroids** |
| --- | --- | --- | --- | --- | --- |
| 1 | D1 | D14 | D18 | D27 | D85 |
| 2 | D1 | D7 | D23 | D43 | D87 |
| 6 | D1 | D3 | D7 | D56 | D51 |
| 8 | D1 | D122 | D161 | D62 | D205 |

irAE: immune-related adverse event; D: Day
