# Supplementary figures and images for: Efficacy and tolerability of anti-programmed death-ligand 1 (PD-L1) antibody (Avelumab) treatment in advanced thymoma
Source: J Immunother Cancer. 2019 Oct 21;7:269. doi: 10.1186/s40425-019-0723-9 (PMC6805423; doi:10.1186/s40425-019-0723-9)

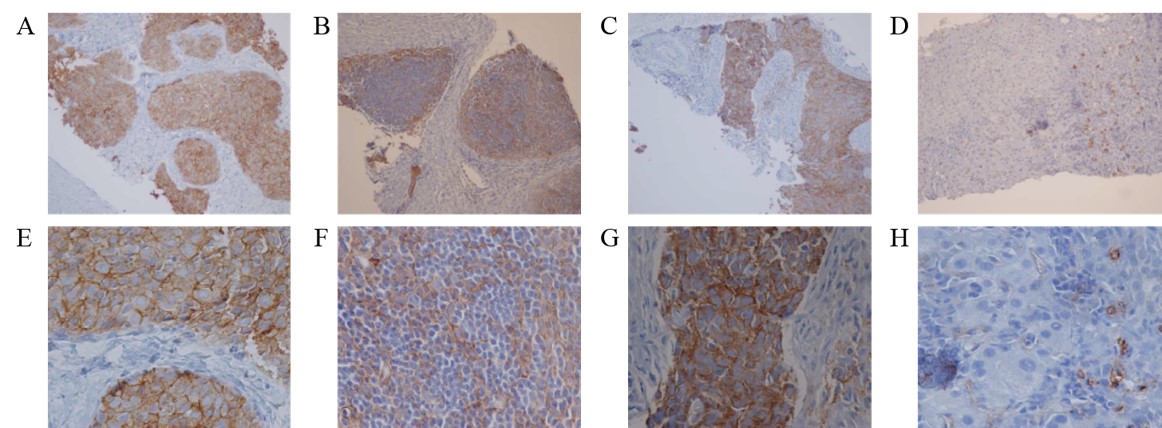

Supplement: Supplementary file 2 — Figure S1. PD-L1 expression in thymoma and thymic carcinoma. (A, E) WHO B3 thymoma with 95% thymic epithelial cell PD-L1 positivity and 3+ intensity. (B, F) WHO B2 thymoma with 50% thymic epithelial cell PD-L1 positivity and 2+ intensity. (C, G) Thymic carcinoma with 90% thymic epithelial cell PD-L1 positivity and 2+ intensity. (D, H) Thymic neuroendocrine carcinoma with focal thymic epithelial cell PD-L1 positivity and 1+ intensity. (A-D: 10x magnification; E-H: 40x magnification.). (JPG 123 kb) [file 40425_2019_723_MOESM2_ESM.jpg]

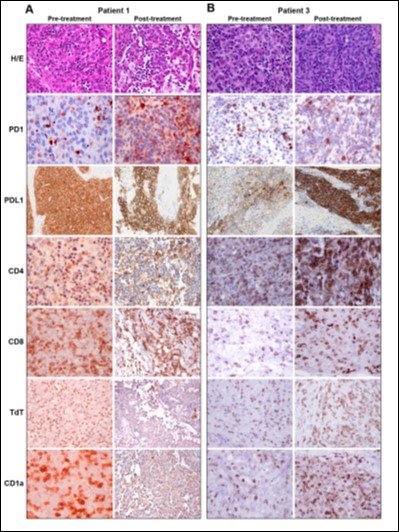

Supplement: Supplementary file 3 — Figure S2. Pre- and post-treatment immunohistochemical evaluation of PD-1, PD-L1, CD4, CD8, TdT and CD1a for patients 1 and 2. PD-1 staining showed scattered PD-1–positive lymphocytes and PD-L1 staining showed diffuse membranous pattern in the epithelial component of the thymoma in both patients. In tissue sections obtained from patient 1 (A), a pre-treatment pleural lesion showed sheets of epithelial cells with abundant well-defined cytoplasm and oval nuclei with a fine chromatin pattern and sparse lymphocytes. Lymphocytes within the tumor expressed CD4, CD8, TdT and CD1a, a pattern consistent with thymocytes. A post-treatment biopsy of a peri-hepatic mass showed morphological characteristics similar to those in the pre-treatment pleural lesion biopsy. However, lymphocytes within the peri-hepatic mass did not express TdT or CD1a; a few CD4 positive cells were seen but the majority of lymphocytes showed only CD8 expression. In tissue sections obtained pre- and post-treatment from patient 2 (B), epithelial cells were seen with abundant well-defined cytoplasm and interspersed lymphocytes. The lymphocytic component in both specimens showed a similar phenotype expressing CD4, CD8, TdT and CD1a consistent with thymocytes. (JPG 82 kb) [file 40425_2019_723_MOESM3_ESM.jpg]

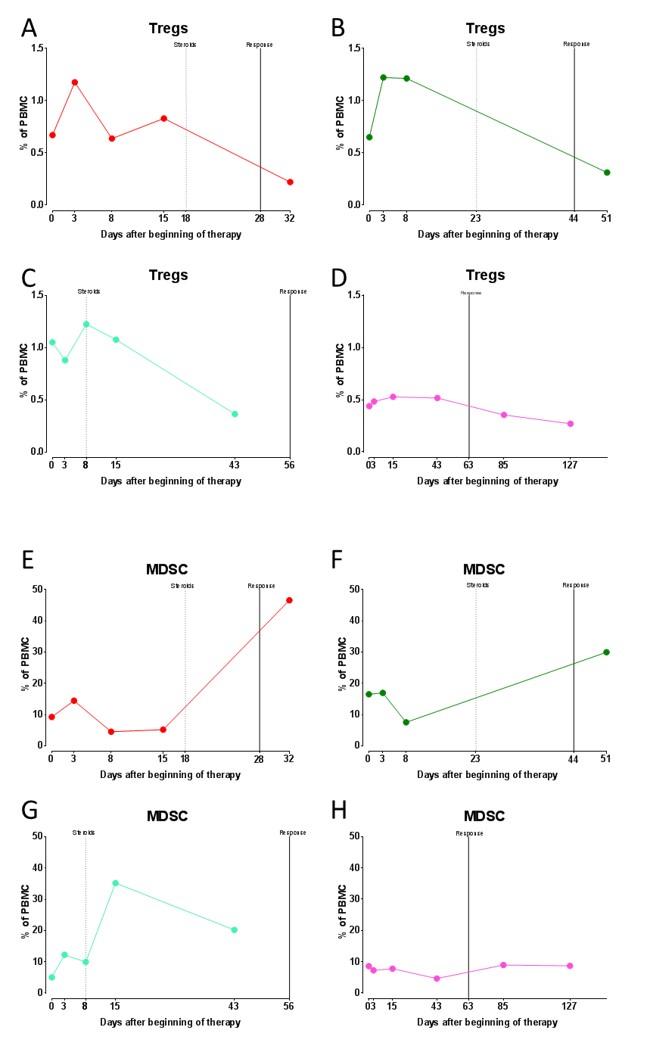

Supplement: Supplementary file 4 — Figure S3. Decrease in regulatory T cells (Tregs) (A-D) and increase in myeloid derived suppressor cells (MDSC) (E-H) following steroids in clinical responders who developed immune-related adverse events (irAEs). Patient 1 (A, E), Patient 3 (B, F), and Patient 6 (C, G) received steroids for irAEs, while patient 8 (D, H) developed clinical response but no irAE for 60 days after documentation of response. Steroids were not used during this time frame. Dashed line denotes timing of steroids and solid line indicates time of clinical response. (JPG 74 kb) [file 40425_2019_723_MOESM4_ESM.jpg]
